# Human Trials on the Prevention of Tunnel Widening by the Emdogain in Anterior Cruciate Ligament Reconstruction

**DOI:** 10.7759/cureus.35960

**Published:** 2023-03-09

**Authors:** Tomoyuki Nakasa, Seiju Hayashi, Atsuo Nakamae, Masakazu Ishikawa, Mitsuo Ochi, Nobuo Adachi

**Affiliations:** 1 Orthopaedic Surgery, Graduate School of Biomedical and Health Sciences, Hiroshima University, Hiroshima, JPN; 2 Orthopaedic Surgery, Hiroshima University, Hiroshima, JPN

**Keywords:** reconstruction, tendon-bone interface, emdogain, tunnel widening, anterior cruciate ligament

## Abstract

Background

Although anterior cruciate ligament reconstruction (ACLR) is an established procedure, some problems remain, such as bone tunnel widening after ACLR. In animal studies, Emdogain (EMD) prevented tunnel widening by promoting tendon-bone healing. This study aimed to evaluate the effects of EMD on the prevention of tunnel widening after anterior cruciate ligament (ACL) injury in humans.

Methods

Nineteen patients who underwent ACLR were included. Seven patients in the EMD group were administered EMDs into the femoral tunnel during ACLR, while 12 patients in the control group were not administered EMDs. After surgery, at two and four weeks and three, six, and 12 months, femoral and tibial tunnel widening were evaluated on computed tomography images. Anteroposterior laxity and clinical scores such as the Lysholm score, the International Knee Documentation Committee (IKDC) subjective form, and the Knee Injury and Osteoarthritis Outcome Score (KOOS) were assessed before surgery and 12 months postoperatively.

Results

Tunnel widening on the femoral side was significantly smaller in the EMD group than in the control group at two weeks. However, there was no significant difference between the two groups at 12 months. There were no significant differences in anteroposterior laxity and clinical scores between the groups before and 12 months after surgery.

Conclusion

EMD administration into the bone tunnel did not prevent tunnel widening at 12 months after ACLR, although tunnel widening of the femoral tunnel was reduced by EMD administration in the early phase.

## Introduction

The anterior cruciate ligament (ACL) is often affected during sports activities, and the number of ACL reconstructions (ACLR) has increased [1]. Although ACLR is a well-established procedure, several cases require revision surgery because of graft failure and recurrent laxity [2]. Several factors have been reported to cause failure, and bone tunnel widening has been recognized as a cause of laxity recurrence. It has been reported that a higher degree of widening correlates with increased anteroposterior laxity as measured by the Lachman test and rotational laxity as measured by the pivot shift test [3]. Because the weakest part during early healing is the attachment between the tendon and bone, the rate of healing and strength of this attachment is crucial for the success of the reconstruction [4,5]. Therefore, the effects of several factors such as growth factors, reagents, and cells have been investigated for tendon-bone healing after ACLR with regard to cell proliferation, extracellular matrix synthesis, and neovascularization, especially in animal models [6,7]. However, these factors have rarely been used clinically because of concerns regarding their possible adverse effects on humans. It is desirable to examine the agents that have been clinically used with an assurance of safety in humans to improve tendon-bone healing after ACLR.

Emdogain (EMD) is made from the enamel matrix proteins of minor porcine teeth and is used as an osteopromotive agent for bone augmentation and regeneration [8]. EMDs stimulate the cellular proliferation and mineralization of osteoblasts and promote the differentiation of osteoblasts and chondrocytes from mesenchymal stem cells [9-12]. With the expectation of osteogenesis in the tendon-bone junction by EMD in ACLR, an animal study using rats revealed the feasibility of EMD in preventing tunnel widening [13]. In that study, EMD improved histological tendon-bone healing at eight weeks and biomechanical healing at both eight and 12 weeks. Based on the excellent results of this animal study, the application of EMD in humans has the potential to enhance tendon-bone repair in ACLR. We hypothesized that the administration of EMD into the femoral tunnel in single-bundle ACLR might prevent tunnel widening and result in good clinical outcomes. This study aimed to evaluate the effects of EMD on tunnel widening by comparing changes in tunnel diameter and clinical outcomes in single-bundle ACLR with or without EMD administration in femoral tunnels in patients with ACL injury.

## Materials and methods

Between April 2015 and April 2016, 19 knees of 19 patients with ACL injuries treated with single-bundle ACLR were included in this study. Informed consent was obtained from all the patients, and those who consented to use EMD were included in the EMD group. Other patients who did not consent to the use of EMD served as the control group. The inclusion criteria were patients older than 16 years who underwent primary single-bundle ACLR with a minimum follow-up period of one year. The exclusion criteria included a history of previous surgeries, including primary ACLR of the knee joint, systemic diseases such as rheumatoid arthritis, and allergic diseases. As a result, seven knees from seven patients in the EMD group and 12 knees from 12 patients in the control group were included in this study. The EMD group consisted of three males and four females, with a mean age of 37.3±15.8 years, and the control group consisted of five males and seven females, with a mean age of 24.8±9.0 years. This study was approved by the local ethics committee of our university and was conducted in accordance with the Declaration of Helsinki and registered with the University Hospital Medical Information Network Clinical Trials Registry (UMIN-CTR ID: 000015497).

Surgical procedure

An autogenous quadrupled semitendinosus tendon was used as a graft. The tendon was connected to the EndoButton CL (Acufex; Smith & Nephew, Manfield, MA, USA) on the femoral side and to the EndoButton tape on the tibial side. A femoral tunnel was created through the far anteromedial portal [14]. The femoral tunnel opening was positioned at the center of the anatomical attachment of the ACL, just behind the resident’s ridge. The passing pin was inserted through the far anteromedial portal and advanced through the lateral cortex at ≥120° of knee flexion. The length of the femoral tunnel was calculated after the diameter was increased to 4.5 mm by an EndoButton drill. Subsequently, a femoral bone socket was created using a headed cannulated reamer with the same diameter as that of the proximal portion of the graft. Remnant-preserving single-bundle ACLR was performed when the diameter of any proximal ACL remnant was more than one-third of the original size and the ACL remnant bridged between the tibia and intercondylar notch or posterior cruciate ligament. The graft composite was passed through the femoral and tibial bone tunnels, and the proximal side of the graft was fixed using the EndoButton CL. The grafts were then fixed onto the tibia with a 50N tension force and two staples (Meira, Nagoya, Japan) with the knee maintained between 20° and 30° flexion. In the EMD group, the space in the tendon-bone interface on the femoral side was filled with 150 μl of commercially available EMD (EMDGAIN; Biora, Malmö, Sweden). In the control group, no reagent was administered to the tendon-bone interface. The reconstructed knee was immobilized using a soft knee brace for two days after surgery. The patients were allowed knee movement exercises with a brace for three days. Partial weight-bearing was started 10 days postoperatively, and full weight-bearing was permitted 17 days postoperatively. Running was allowed 4.5 months after surgery, and sports training, such as jumping, was allowed six months postoperatively.

Evaluation

Clinical evaluations were performed before and 12 months after surgery. Side-to-side differences in anterior knee laxity were evaluated using a Kneelax 3 Arthrometer (Monitored Rehab Systems, Haarlem, Netherlands). The validated patient-reported outcome measures of the Lysholm knee scoring scale [15], five subscales of the Knee Injury and Osteoarthritis Outcome Score (KOOS) [16], and the International Knee Documentation Committee (IKDC) subjective form [17] were used for clinical evaluations.

**Figure 1:**
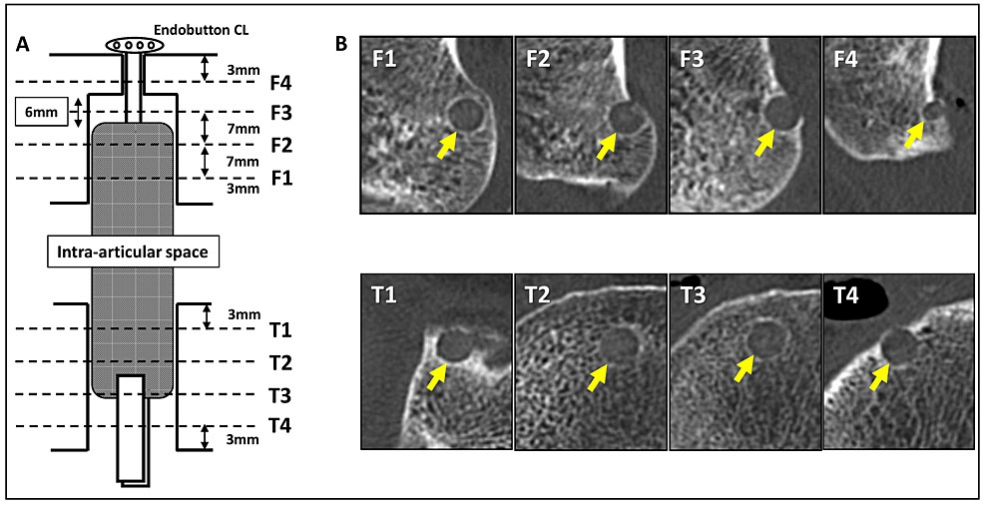
Evaluation of the tunnel widening. (A) Schema of the regions in which tunnel diameters were measured. (B) Representative images of bone tunnels by computed tomography. Arrows indicate the bone tunnel in each region.

Tunnel widening was evaluated using computed tomography (CT) imaging after surgery. A 64-multi-detector-row CT (VCT; GE Medical System, Milwaukee, WI, USA) was used. Scanning parameters included a gantry rotation speed of 0.6 seconds/rotation, 1.25 mm collimation width × 16 detectors, CT pitch factor of 0.562, and field of view of 25-30 cm. The CT dose index (CTDI) volume was 7.67 mGy. Four axial slices perpendicular to the bone tunnels were created on the femoral and tibial sides using commercially available software (Virtual Place Raijin; AZE, Ltd., Tokyo, Japan). Femur and tibia sides were divided into four regions (femur: F1, F2, F3, and F4; tibia: T1, T2, T3, and T4), and the tunnel diameters of these regions at each point were measured at two days, two weeks, and four weeks and three, six, and 12 months postoperatively (Figure 1). The tunnel diameter at each time point was divided by the tunnel diameter at two days postoperatively to calculate the tunnel widening rate.

Statistical analysis

Statistical differences between the two groups were calculated using the Student’s t-test. Statistical significance was set at P<0.05.

## Results

**Figure 2:**
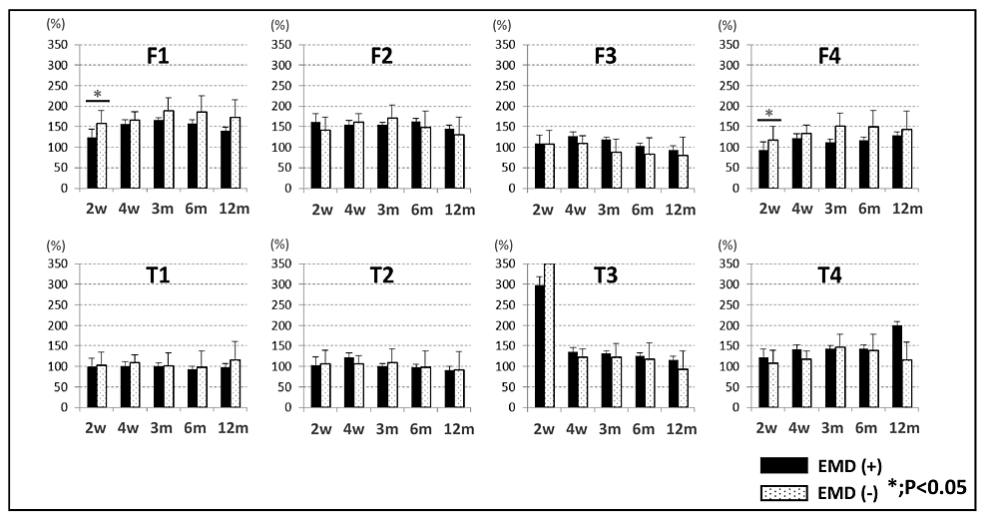
The tunnel widening ratio in the femoral and tibia at two and four weeks and three, six, and 12 months. EMD: Emdogain, w: weeks, m: months *P<0.05

The mean body mass index in the control group and EMD group was 23.2±4.0 kg/mm^2^ and 24.4±2.3 kg/mm^2^, respectively, and there was no significant difference between both groups. Bone tunnel widening on the femoral and tibial sides was observed in all patients. In the femoral tunnels, the tunnel widening ratios of F1 and F4 in the EMD group at two weeks were significantly lower than those in the control group (P<0.05). In the tibial tunnels, bone tunnel widening was observed at T3 and T4 in both groups. In all regions of the femur and tibia, there was no significant difference in the tunnel widening ratio between the two groups at 12 months (Figure 2).

In the clinical outcomes, the preoperative side-to-side difference in Knee Lax 3 was 3.6±2.9 mm in the control and 4.6±1.5 mm in the EMD group, and there was no significant difference. Both groups had significantly improved side-to-side differences of the Knee Lax at one year postoperatively, and there was no significant difference between both groups at one year postoperatively. The Lysholm, KOOS, and IKDC scores were significantly improved from preoperative to one year postoperatively in both groups, and there was no significant difference between the two groups preoperative and one year postoperatively (Table 1). No adverse effects were observed following EMD administration.

**Table 1:** Clinical outcomes in the control and EMD groups. EMD: Emdogain, QOL: quality of life

Preoperative and 12-month operative anteroposterior laxity and clinical scores		Control (n=12)	EMD (n=7)	P value
Anteroposterior laxity	Preoperative	3.6±2.9	4.6±1.5	0.764
	12 months	0.6±1.4	0.4±1.5	0.719
Lysholm score	Preoperative	75.5±13.2	72.4±12.7	0.667
	12 months	91.2±8.9	89.6±13.8	0.96
IKDC subjective	Preoperative	79.4±21.9	82.7±11.5	0.905
	12 months	96.1±4.8	93.2±8.0	0.471
KOOS pain	Preoperative	79.4±21.9	82.7±11.5	0.905
	12 months	96.1±4.8	93.2±8.0	0.471
Symptom	Preoperative	76.6±18.1	74.9±8.8	0.667
	12 months	86.0±15.5	87.3±17.2	0.555
Activity	Preoperative	89.5±10.4	88.2±8.7	0.897
	12 months	98.7±2.4	98.7±3.3	0.728
Sports	Preoperative	51.0±28.2	48.0±22.0	0.952
	12 months	93.2±6.0	90.7±11.0	0.96
QOL	Preoperative	49.4±28.3	54.2±24.1	0.667
	12 months	82.4±13.6	87.5±13.7	0.484

## Discussion

This study revealed that only early tunnel widening in the femoral tunnel could be prevented by EMD administration, but there was no significant difference in tunnel widening of both femoral and tibial tunnels with and without EMD at 12 months and clinical outcomes with and without the administration of EMD into the bone tunnels during ACLR, although EMD has the capability to enhance biomechanical tendon-bone healing. Tunnel widening has been reported to affect both biological and mechanical features [18]. In our study, it was thought that the mechanical features might be stronger than biomechanical healing by EMD.

However, the relationship between bone tunnel widening and clinical outcomes remains controversial. No correlation has been reported between bone tunnel widening and clinical outcomes in patients [18-21]. In contrast, Järvelä et al. demonstrated that a higher amount of widening is correlated with increased anteroposterior and rotational laxity [3]. It has been reported that the bone tunnel created during ACLR is markedly enlarged within six weeks postoperatively and tends to enlarge up 1-3 years postoperatively [22]. Since there is the possibility that anteroposterior and rotational laxities will occur due to tunnel widening in the long-term follow-up, minimizing the duration of exposure to mechanical or biological factors is a reasonable treatment.

To improve bone and tendon healing, various biological augmentations, including growth factors, mesenchymal stem cells, pharmaceuticals, and gene therapy, have been employed in animal models [23]. Overall, these biological augmentations enhanced healing at the graft-tunnel interface in animal models. However, some of these have not been tested for their safety in humans. Several clinical studies have evaluated platelet concentrates and bone marrow-derived mesenchymal stem cells (MSCs). However, it has been reported that platelet concentrates and bone marrow-derived MSCs have no beneficial effects on the prevention of tunnel widening [24]. In studies of biological augmentation for tunnel widening, approximately 50% of these studies reported positive results, but the actual effectiveness of biological augmentation for the prevention of tunnel widening is still unclear because there is a mixture of various techniques such as remnant repair and bone substitute and no difference in clinical outcomes [24].

In our study, EMD was administered into the bone tunnels during ACLR. EMD is a mixture of enamel matrix derivatives obtained from porcine teeth [8]. The major component of EMD is amelogenin, a member of the hydrophobic protein family [25]. Other components of EMD include enamelin, tuftelin, tuft proteins, ameloblastin, and extra peptides such as bone morphogenetic proteins (BMPs) and transforming growth factor-beta [26]. EMD is widely used in periodontal treatments to promote bone formation in the clinical setting [25]. EMD has the ability to stimulate cell proliferation and mineralization of both preosteoblasts and osteoblasts and induce the differentiation of MSCs into osteoblasts and chondrocytes [9-12]. Because of these characteristics and safety in clinical use, Kadonishi et al. examined the efficacy of EMD administration on tendon-bone healing of the tunnel in ACLR in a rat model. They demonstrated that collagen fibers connected to the bone at the tendon-bone interface were increased histologically, and the failure load of the graft was increased compared to that in animals without EMD administration [13].

We administered EMD to the femoral bone tunnels during ACLR in humans, and tunnel widening of the femoral tunnel at two weeks was prevented; however, there were no significant differences between patients with and without EMD at 12 months. Since the postoperative immobilization period at our institution was two days, there is the possibility that micromotion, called the “bungee-cord” or “windshield-wiper” effect, might have affected the tunnel enlargement before tendon-bone healing was achieved. In addition, EMD was administered only to the femoral bone tunnel because of the difficulty of administering EMD to the tibial tunnels. In the current study, a suspension device was used to fix the graft, which may have induced micromotion in the bone tunnels. Since the effect of EMD on the prevention of tunnel widening in the femoral tunnel in the early phase was observed, the administration of EMD into both the femoral and tibial tunnels might have prevented tunnel widening at 12 months postoperatively by reducing the micromotion of the graft within the tunnels from the early phase. The influence of mechanical factors on the tendon-bone interface may be greater in humans than in animals. A combination of surgical techniques that prevent micromotion in bone tunnels and EMD administration may need to be used to evaluate the efficacy of EMD administration.

This study has several limitations. First, the sample size was small. Although the safety of EMD has been confirmed, some patients refused to participate. In addition, the mean age of the EMD treatment group was relatively higher than that of the nontreatment group, which may have affected the bone quality of the knee joint. Further studies with larger sample sizes are warranted. Second, a histological evaluation was not performed. In animal studies using EMD, the promotion of bone-tendon healing was histologically observed. In humans, the tendon-bone interface should be evaluated histologically; however, because this is not ethically feasible, clinical scores and tunnel enlargement on CT were assessed. Finally, the evaluation of tunnel enlargement using CT was only performed for up to 12 months. Because it has been reported that tunnel widening progresses until three years postoperatively, CT evaluation should be performed in the long term [22]. In contrast, Ugutmen et al. reported that tunnel enlargement progressed until six months postoperatively, at which time the enlargement stopped progressing, and the tunnel diameter slightly decreased at the two-year follow-up [27]. Because it is controversial how long tunnel enlargement continues, it is difficult to decide how long to perform a CT scan. In addition, radiation exposure is a problem when performing CT scans.

## Conclusions

The administration of EMD into the bone tunnel did not prevent tunnel widening after ACLR at 12 months, although tunnel widening of the femoral tunnel was reduced by EMD administration at two weeks. The tunnel widening exhibited both biological and mechanical features. In humans, mechanical factors may be greater than biological factors. In addition to EMD administration, mechanical factors may prevent bone tunnel widening.
